# 1391. Body Mass Index and Leptin Levels at Different Stages of the Tuberculosis Spectrum

**DOI:** 10.1093/ofid/ofab466.1583

**Published:** 2021-12-04

**Authors:** Wajih Askar, Manuel G Feria, Shinsmon Jose, Rajat Madan, Moises A Huaman

**Affiliations:** 1 University of Cincinnati/Department of Infectious Disease, Cincinnati, Ohio; 2 University of Cincinnati, Cincinnati, Ohio

## Abstract

**Background:**

Leptin is an adipose tissue-derived cytokine that plays a role in energy regulation and immune functions. High leptin levels and obesity have been associated with decreased risk of developing active TB. We aimed to characterize the association between body mass index (BMI) and leptin levels in patients at different stages of tuberculosis (TB).

**Methods:**

Data from a cross-sectional cardiovascular risk study of 40 to 70 years old individuals enrolled in Lima, Peru, and Cincinnati, US, were analyzed. Four categories based on TB and treatment status were defined: no TB infection (QuantiFERON-TB test negative; n= 31), latent TB infection (LTBI; QuantiFERON-TB test positive; n= 43), active TB on treatment (in the continuation TB treatment phase; n= 30), and post-TB (within one year of TB treatment completion; n=16). BMI and plasma leptin levels were compared among the four groups using the Kruskal-Wallis test, followed by Dunn’s multiple comparison test if differences were found in the Kruskal-Wallis test. Multivariate ordered logistic regression models were used to assess factors associated with leptin levels, adjusted for potential confounders.

**Results:**

The median age was 53 years, and 51% were female. BMI was different between study groups (p< 0.01), with LTBI individuals having the highest BMI compared to other groups; see Figure 1A. Leptin levels were marginally low in the group with active TB on treatment, but no significant differences were found between groups (p=0.44; see Figure 1B). In multivariate analysis, leptin was associated with female sex (OR 23, 95%CI, 9-58), BMI (OR, 1.5, 95%CI, 1.2-1.7), and coronary plaque ≥25% stenosis (OR, 0.29, 95%CI, 0.08-0.99). Body mass index (BMI) and plasma leptin levels in participants with negative QuantiFERON-TB test (QFN-), latent tuberculosis infection (LTBI), active tuberculosis on treatment (ATBT), and post-TB treatment (TB-treated).

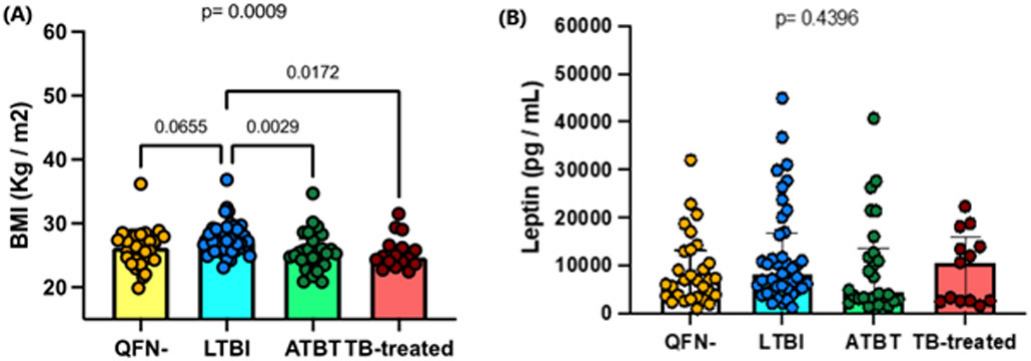

Significance was determined using the Kruskal-Wallis test, followed by Dunn’s multiple comparison test if the Kruskal- Wallis test p-value was <0.05.

**Conclusion:**

LTBI individuals had a higher BMI compared to persons with active TB on treatment and post-TB. Higher leptin levels were associated with higher BMI, but we found no association between leptin and TB status in our cohort.

**Disclosures:**

**All Authors**: No reported disclosures

